# Retrospective analysis of mature cystic teratomas in a single center and review of the literature

**DOI:** 10.4274/tjod.86244

**Published:** 2018-06-21

**Authors:** Çiğdem Yayla Abide, Evrim Bostancı Ergen

**Affiliations:** 1 İstanbul Zeynep Kamil Women and Children’s Diseases Training and Research Hospital, Clinic of Obstetrics and Gynecology, İstanbul, Turkey

**Keywords:** Teratoma, CA19-9, adnexal masses

## Abstract

**Objective:**

The aim af this study is to evaluate patients with mature cystic teratomas (MCT) with regard to the view of updated knowledge using our retrospective findings.

**Materials and Methods:**

This was a retrospective study and included a total of 306 patients from 2013 through 2017 at the İstanbul Zeynep Kamil Women and Children’s Diseases Training and Research Hospital.

**Results:**

The mean age of the patients was 34.03±11.98 years. Thirty (9.8%) patients were postmenopausal. Torsion was detected in 17 (5.6%) patients. There was a statistically significant relationship between MCT and CA 19-9 levels in our series (p<0.01) but no statistically significant correlation was found with other markers.

**Conclusion:**

The possibility of malignancy at postmenopausal ages and in large MCT should not be forgotten. It should be kept in mind that MCT can be seen in unexpected places.

**PRECIS:** Mature cystic teratomas has association with CA19-9.

## Introduction

Mature cystic teratoma (MCT) is the most common ovarian tumor in women aged 20-30 years and accounts for 95% of ovarian teratomas^(1)^. Most MCTs are unilateral and benign. Only 10-17% of cases are bilateral^(2)^. MCTs include tissues of ectodermal (eg., skin, hair follicles, sebaceous glands), mesodermal (eg., muscle, urine) and endodermal origin (eg., lung, gastrointestinal)^(3)^. These tumors have a characteristic ultrasonic image and the specificity of ultrasound is 98-100%^(4,5)^. Malignant transformation is detected in 0.17-2% of MCTs. When it occurs, the most common is squamous cell carcinoma^(6)^. Risk factors for malignancy, age over 45 years, tumor size 10 cm and larger, rapid growth, and imaging findings (eg., low resistance tumor flow in Doppler)^(2,7)^. Early diagnosis and treatment of malignant transformation of MCT is very important because 5-year survival is only 15-30%^(8)^. We retrospectively analyzed preoperative, postoperative outcomes, and the clinicopathologic characteristics of mature cystic teratomas of the ovary at our institution.

## Materials and Methods

This was a retrospective study of all MCTs from 2013 through 2017. The data were obtained from the hospital database of İstanbul Zeynep Kamil Women and Children’s Diseases Training and Research Hospital. It included a total of 306 cases. Data regarding age, size, laterality, gross morphologic features, levels of tumor markers alpha fetoprotein (AFP), cancer antigen (CA) 19-9, CA 15-3, carcinoembryonic antigen (CEA), and CA 125, complications, and surgery performed were retrieved from the hospital archives. A laparoscopic approach was preferred for patients who had no contraindications for laparoscopic surgery such as cardiac or pulmonary diseases, and no contraindications for being placed in the lithotomy position, but mainly the surgeon determined which surgery was to be performed.

### Statistical Analysis

The IBM SPSS Statistics 22.0 program was used for the statistical analyses. When the study data were evaluated, the relationships between descriptive statistical methods (mean, standard deviation) was used. Besides these, Student’s t-test was used for the comparison of two groups with normal distribution, and Spearman’s correlation analysis was used to examine parameters without normal distribution. The chi-square test, Fisher’s exact test, and Yates’s continuity correction test were used for the comparison of qualitative data. Significance was assessed at p<0.05 level.

## Results

The mean age of the patients was 34.03±11.98 years. Thirty patients (9.8%) were postmenopausal. The symptoms of the patients and the imaging methods used can be seen in Table 1. Torsion was detected in 17 (5.6%) patients. No cyst rupture was observed in any of the patients. Cyst diameters ranged from 1 to 20 cm with a mean of 6.68±6.0 cm. The mean CA 125 level was 22.96±15.5 U/mL, the mean CA 19-9 level was 71.11±17.8 U/mL, the mean CA 15-3 level was 13.97±13.5 U/mL, the mean CEA level 1.39±1.09 ng/mL, and the mean AFP level was 177.32±1.9 ng/mL. The normal values for CA 125 is 0 to 35 U/mL. The reference range of serum CA 15-3 is less than 30 U/mL. The reference range of serum CA 19-9 is less than 37 U/mL. The normal range for CEA in an adult non-smoker is <2.5 ng/mL, and for a smoker it is <5.0 ng/mL. An AFP level of less than 10 ng/mL is normal for adults. There was a statistically significant correlation between cyst diameter and CA 19-9 (p<0.01). As the diameter of the cyst increased, CA 19-9 level also increased. There was no statistically significant correlation between cyst diameter and other marker levels (p>0.05). When the distribution of localization was examined, 25 (8.2%) patients had bilateral localization and 281 (91.8%) had unilateral localization. Laparoscopic surgery was performed in 57.8% (n=177) of patients. In 19 (6.2%) patients, cyst rupture was detected during surgery, but none of the patients had chemical peritonitis in the post-operative period. Abundant lavage was applied to patients in whom rupture had occurred. Ninety-one cases (29.7%) were frozen and 9 cases (2.9%) were malignant. Three were reported as immature teratomas, three as squamous cell carcinoma, two as yolk sac tumor, and one as adenocarcinoma. The location of the MCTs and summary of the surgical procedures is presented in Table 2. The mean postoperative hospital stay was 1.89±1.06 days. Six (2%) patients had recurrence after 1 year. A dermoid cyst had been discovered during cesarean section in 8 patients.

## Discussion

MCTs are usually diagnosed at reproductive ages and treatment is surgical. In this study, we observed an association with CA 19-9 and MCT, and we present 2 cases of MCT in unusual locations. Consistent with the literature, the mean age in our study was 34.03±11.98 years. In previous studies, it was found to present most commonly between the ages of 20-30 years^(9)^. In our study, we found that 91.8% of the MCTs were unilateral and more frequent on the left side (47.7%). The literature is unanimous with regards the unilaterality of the tumors, and there is no consensus on the right and left dominance. Some studies reported them more frequently on the right, some on the left^(2,10,11,12)^. Some 38.2% of the patients were admitted to hospital with symptoms of pain, and 40.2% cases were found incidentally. In parallel with the literature, MCTs could be detected in 16.6% and 75% of asymptomatic patients during routine physical examination and during any pelvic operations, respevtively^(1,13,14,15,16)^. The mean size of tumor was 6.68±6.0 cm. This is in accordance with previous studies where 60% of tumors were 5-10 cm in diameter^(10,15,16,17)^. Similar to previous studies, in our study, the most common complication of MCT was torsion and its detection rate was 5.6%. Malignant transformation of MCT was 2.9%. In the literature, malignant transformation is found between 1 and 3% and torsion rates are between 3.5 and 9.2%^(6,13,14)^. As supported by the literature, advanced age and tumor size are evaluated as risk factors for malignant transformation^(2,7)^. In our study, the mean diameter of MCTs with malignant transformation was 11.23 cm, and the mean age of patients with MCT with malignant transformation was 47 years. There was a statistically significant relationship between MCTs and CA 19-9 levels in our series (p<0.01) but no statistically significant correlation was found with other markers. In the literature, Ito^(18)^ reported that MCTs has an association with CA 19-9 levels. However, Chen et al.^(19)^, in contrast to our study, found that Ca 125, CA 153, and AFP together had an association with MCTs. With this study, we also evaluated 2 cases MCT that were rare in terms of their location. A 22-year-old patient presented with a vaginal mass of approximately 11 cm. It was filled with sebum and hair, and was diagnosed as MCT by the pathologists. Vural et al.^(20)^ in 2015 concluded that the presence of vaginal teratoma was rare; only 8 cases have been reported worldwide. Our case was the largest of these cases. In another case, a 44-year-old woman was admitted to our hospital with a sebaceous cyst of about 3 cm in diameter in the labium majus, which was diagnosed as MCT in the pathology report. The rate of rupture during laparoscopy is very high in MCTs. Approximately 54% has been detected^(21,22,23)^. The probability of rupture has been found to be independent of size and location of MCT^(24,25)^. Cyst rupture was detected during surgery in 19 cases in our series in agreement with these studies. All cases of cyst rupture occurred during laparoscopy. However, we detected no chemical peritonitis postoperatively.

### Study Limitations

The number of patients is small because it is a single-center experience.

## Conclusion

The possibility of malignancy in women of postmenopausal age and in large MCTs should not be forgotten. It should be kept in mind that MCTs can be seen in unexpected places such as the eyelids, mouth, vagina, and labia majus. We observed an association with CA 19-9 and MCT, but further studies are needed.

## Figures and Tables

**Table 1:**
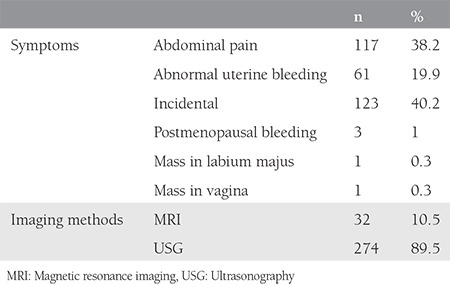
Symptoms and imaging methods of the patients

**Table 2:**
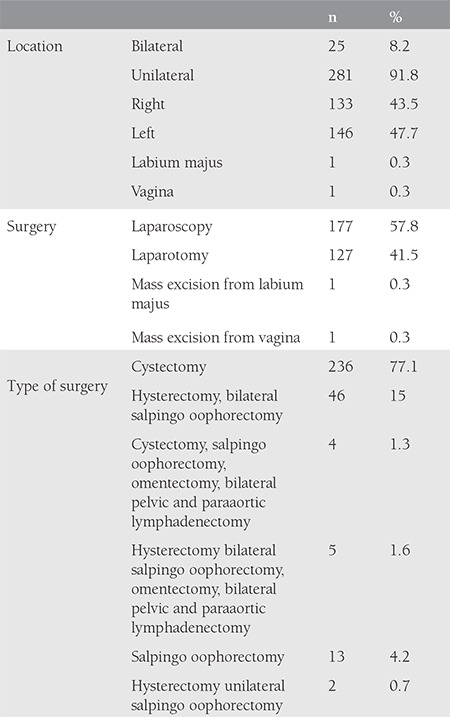
Localization of mature cystic teratomas and summary of the surgical procedure utilization
